# Meta-analysis of mortality factors after COVID-19 infection in pediatric oncology patients

**DOI:** 10.3389/fonc.2025.1594617

**Published:** 2025-08-06

**Authors:** Qingchen Liu, Xia Wang, Xueqin Zhou

**Affiliations:** ^1^ People’s Hospital of Fengjie, Chongqing, China; ^2^ Gynaecology, People’s Hospital of Fengjie, Chongqing, China; ^3^ Department of Gynecology and Obstetrics, People’s Hospital of Fengjie, Chongqing, China

**Keywords:** pediatric tumors, COVID-19, mortality factors, prognosis, meta-analysis

## Abstract

**Objective:**

There are few clinical studies related to COVID-19 in pediatric cancer patients, and systematic reviews or meta-analyses on its mortality risk factors are particularly lacking. Therefore, we conducted this meta-analysis to systematically analyze the mortality risk factors of pediatric cancer patients after COVID-19 infection, providing effective evidence-based medical evidence for epidemic prevention and control and clinical treatment of pediatric COVID-19 patients.

**Methods:**

Electronic databases of PubMed, Embase, Cochrane Library and Web of Science were searched using “cancer” “COVID-19” “children” “mortality” related subject headings and keywords. The Newcastle-Ottawa Scale (NOS) was used to assess the quality of the included studies. Outcomes included age, weight, clinical complications in patients, cancer type, consolidation of cancer treatment, and critical illness. The quality of observational studies was assessed using the Newcastle-Ottawa Scale, which includes criteria such as study population selection, comparability, and evaluation of exposure or outcome, by two independent reviewers.

**Results:**

A computerized search of the literature yielded six observational studies with a total of 2,696 patients, and a pooled assessment of predictive factors revealed that the occurrence of adverse clinical complications, the presence of solid tumors, and the presence of acute and critical conditions significantly increased mortality in pediatric oncology patients (P < 0.05), although, overall, aggressive consolidation of cancer treatment significantly reduced the death of patients. Although overall,being in the cancer consolidation treatment period is significantly associated with a reduced risk of patient mortality, there is still an increase in mortality with Radiotherapy, possibly due to immunocompromise (P < 0.05), whereas Immunotherapy and Surgery do not affect patient prognosis. Subgroup analyses showed that prolonged consolidation of cancer treatment reduced mortality. The sensitivity analysis of the results of the outcome indicators was stable with low sensitivity and high confidence.

**Conclusion:**

Adverse clinical complications, the presence of solid tumors, and the occurrence of critical conditions increase mortality in pediatric cancer patients. Receiving aggressive cancer treatment is associated with lower mortality rates, but this association should be interpreted with caution, as it may be confounded by other factors.

**Systematic Review Registration:**

https://www.crd.york.ac.uk/PROSPERO/, identifier CRD420250570932.

## Preamble

The novel coronavirus(COVID-19) infection pandemic has overwhelmed health care systems worldwide (1), with approximately 419 million people infected with COVID-19 disease, resulting in more than 5.8 million deaths since its emergence in 2019 (2). Currently, covid have severely impacted the treatment of cancer patients (3). During the covid pandemic, cancer deaths increased as cancer screening was drastically reduced and ongoing or planned treatments were delayed (42). In addition, it has been reported (4, 5) that immunosuppressive therapy for cancer patients weakens the body’s immune system, so cancer patients infected with covid are at increased risk of exacerbations. Complications of infection with covid, such as acute respiratory distress syndrome and impaired mechanical ventilation, may also lead to poor quality of survival.

Reports indicate that infections in children are less severe, but may also have serious consequences, including poly inflammatory syndrome, hospitalization requiring mechanical ventilation, and death (6). The severity of infections in children is associated with a variety of risk factors, especially those with immunosuppressive diseases(cancer, lung disease, congenital heart disease, obesity, and diabetes) (7–9).The risk of infection and severity of disease varies depending on the presence of risk factors, which include age, comorbidities, immunosuppressive status, and appropriate use of nonpharmacologic measures (6).

COVID-19 is more prevalent in children with cancer than in the general pediatric population (10). These patients have an increased likelihood of infection, and morbidity and mortality are elevated. In addition, oncology treatment and follow-up require frequent visits, and immunocompromised high-risk cancer patients have increased exposure to hospital and healthcare personnel, which may further increase the risk of infection (11). However, researchers have also found that a reduced inflammatory response in patients with blood cancers may protect them from the serious harms of COVID-19 (12, 13). Therefore, understanding the clinical characteristics of pediatric COVID-19 patients is of great significance for the early identification and effective treatment of pediatric infected patients. However, there are few clinical studies related to pediatric COVID-19 and most published studies of pediatric COVID-19 beneficiaries have small sample sizes (14–19),and emerging evidence is limited to case reports, case series, and small cohorts, mostly from developed countries, and there is only one meta analysis (20). Therefore, in this study, we systematically analyzed the risk factors for eventual death after COVID-19 infection in pediatric oncology patients by Meta-analysis to provide a reference for the prevention and control of the epidemic and the clinical management of pediatric COVID-19 patients.

## Methods

### Search strategy

Computerized search for observational studies related to risk factors for eventual mortality after COVID-19 infection in pediatric oncology patients from November 1, 2019 until August 2024 using PubMed, Base, Web of science, Cochrane library. accessed for pediatric oncology patients who developed COVID-19 infection after Studies related to mortality, accessed from the above databases’ builds until August 2024, using MESH English keywords such as “cancer” “COVID-19” “children” “mortality”, etc., and no filters were used. The search was limited to full text and no language or geographic restrictions were imposed on the search. In addition, we screened the references of the included studies for any other relevant studies.

### Inclusion and exclusion criteria

Inclusion criteria: The study subjects are COVID-19 patients aged 19 years and younger with malignant tumors who have clear records in each hospital or organization; The types of studies are clinically relevant observational studies, including retrospective case-control, retrospective cohort studies, prospective case-control, and prospective cohort studies, which illustrate the effect of various indicators on mortality in the patients and are published in peer-reviewed scientific journals and Conference published studies; Observational outcome indicators are age, weight, clinical complications in patients, cancer type, cancer consolidation treatment and critical illnesses, and the effect of different solid tumors and different consolidation treatments on patients’ mortality; Research reports published in English.

Exclusion criteria: Studies on non-COVID-19 infections in pediatric tumors; Overlapping with other interventions; Study data could not be extracted and were not related to mortality; Review, case study, literature study, Meta-analysis, etc.; Repeatedly published literature; Studies were not approved; Studies could not be obtained due to copyright issues.

### Literature screening and quality assessment

To ensure the accuracy and completeness of data extraction, we have established a standardized data extraction process. Data extraction was performed by two systematically trained independent reviewers using a uniformly designed Microsoft Excel data extraction form. The natural data extraction form was pre-tested and revised several times and contained the following main modules: including participant data, inclusion and exclusion criteria, intervention details and outcome measures. Results from two reviewers were compared and any discrepancies or differences were resolved through discussion with a third reviewer who evaluated the same data. Study authors were contacted for additional information to determine the final literature to be included, if necessary. Data were extracted from the literature screened above, including the last name of the first author, year of publication, patient characteristics and study characteristics, risk factors, and clinical outcomes of interest, and were made into a trilinear table.

During the data extraction process, we developed detailed processing principles: for multiple publications of the same study, the most recently published version or the version with the most complete data was preferred as the primary data source; when specific values for survival analysis were not provided directly in the literature, we used the validated Engauge Digitizer 4.1 tool (40) to extract survival data from Kaplan-Meier survival data from Kaplan-Meier curves, and data extraction was performed independently by two investigators to ensure accuracy. All extracted data were cross-checked, and for items with disagreement, a third senior investigator arbitrated and consensus was reached through group discussion. In addition, we established a data verification mechanism to randomly select 20% of the included studies for review to ensure the reliability of data extraction. All extracted data were entered into a standardized database and double entry was used to ensure data accuracy and provide a high-quality data base for subsequent Meta-analysis.

The quality of observational research literature was assessed by the researchers based on the Newcastle-Ottawa Scale (NOS) (41) to evaluate the methodological use of the included literature. The scale is divided into 2 sections for evaluating cohort studies and case-control studies, and each section has 3 columns (8 entries in total) for study population selection, comparability, and exposure or outcome evaluation. During the assessment process, we should strictly adhere to the fact that if an item falls into the * criteria in the assessment, it will be counted as 1 point, and the maximum score is 9 points. Cumulative scores of 5 or more will be rated as high-quality literature and can be included in the meta-analysis, if the score is below 5, it will be rated as low-quality literature and will not be included.

### Statistical analysis

Meta-analyses of extracted data were analyzed using RevMan 5.4.1 provided by the Cochrane Collaboration. Meta-analyses and individual study estimates were presented as forest plots. The outcome metrics for this study were the calculation of 95% confidence intervals for odds ratios (OR, odds ratio) and hazard ratios (HR, hazard ratio) for dichotomous variable data, combining HR values using the inverse variance method, and calculating 95% confidence intervals (CI). All outcome data were processed using a random effects model. Heterogeneity between studies was assessed using chi-square and I2 tests. Studies were considered homogeneous if P > 0.1 and I2 ≤ 50%; conversely, studies were heterogeneous if P ≤ 0.1 and I2 > 50%, and sensitivity analyses and subgroup analyses were performed on the sources of heterogeneity and factors affecting the posterior values of the combined effects. In the presence of heterogeneity, subgroup analyses were performed to explore sources. Sensitivity tests were used to determine whether analytic heterogeneity would affect the stability of the results by comparing the effect sizes of the random-effects model and the fixed-effects model. To identify potential publication bias and small-sample effects, we used a multidimensional assessment method: first, the symmetry of the funnel plot was assessed by the visual method, focusing on the distribution of the small-sample studies at the bottom of the graph, with small sample sizes and low study precision at the bottom of the funnel plot, dispersing around; and with large sample sizes and high study precision at the top of the funnel plot, concentrating toward the middle. The significance level was set at α = 0.05.

## Results

### Literature search and study identification

**Figure 1:**
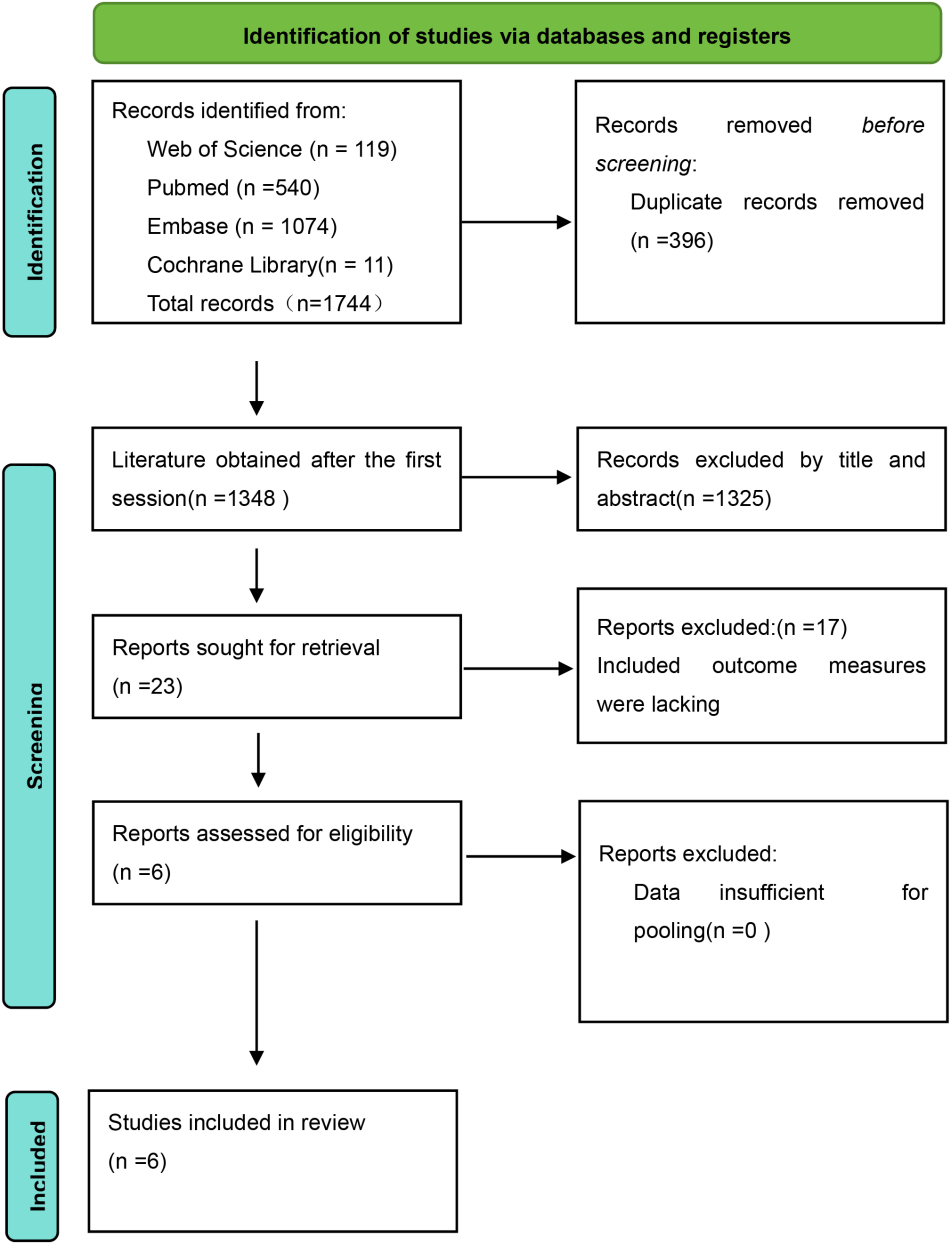
Revman search flowchart.

A total of 1744 studies were retrieved in our initial search, of which 540 were PubMed, 119 Web of Science, 11 Cochrane Library, and 1074 Embase, and we identified 1348 records after removing duplicates of 396 items. The full text of 23 articles was subsequently reviewed based on screening of titles and abstract reviews. Six (14–16) reports of observational studies that met the inclusion criteria were ultimately eligible for data extraction and quantitative analysis (**Figure 1**).

### Results of study characterisation

A total of six recently published (2021-2023) literatures (14–19) were included in this study, evaluating a cumulative total of 2696 pediatric tumor patients. Since the study (14) was conducted by the Global Study on Noncommunicable Diseases in Children without specific authors, “Collaborators 2022” is used as a substitute name. Characterization of the studies revealed the following: all studies reported the age range of patients (median age 6.0-9 years), covering the population from neonates (8 days) to adolescents (18 years). Four studies (14–16, 19) specified the gender distribution, with 59.0%-67.7% males, and the overall male-to-female ratio was close to 1.3:1.Cancer type: five studies (14–17, 19) classified tumor types in detail, with hematological malignancies predominating (leukemia, lymphoma, 69%-85.5%), followed by solid tumors (neuroblastoma, sarcoma, etc., 6%-15%). TREATMENT AND FOLLOW-UP: All studies used multimodal treatment including chemotherapy (85.5%), radiotherapy (6.2%-8.3%), immunotherapy and bone marrow transplantation, with some patients receiving palliative care (4.5%). Follow-up spanned 1-18 months, with 3 studies14,16,19 following for more than 6 months and the longest follow-up up to 18 months. CLINICAL OUTCOMES: Mortality within 30 days was significantly associated with COVID-19 infection, and children with low BMI had a poorer prognosis. Early intensive therapy (e.g., induction/consolidation chemotherapy) reduces the risk of death, but requires vigilance for complications of severe infections (**Table 1**).

**Table 1:** Table of basic characteristics of the included literature.

Study (year)	Sample characteristics	Cancer type	Primary treatment	Duration of follow-up	Key outcomes
Collaborators 2022 (14)	Age: median 6 years (IQR 3-11) Sample size: 2108 (59.0% male)	Hematologic malignancies (acute lymphoblastic leukemia, non-Hodgkin’s lymphoma, Hodgkin’s lymphoma, etc.) Solid tumors (neuroblastoma, sarcoma, etc.)	Chemotherapy, Radiotherapy, immunotherapy	30 days to 12 months	Prompt treatment reduces risk of death within 30 days
Ana Luiza Magalhães de Andrade-Lima 2023 (15)	Age: 6.8 years (range June-18) Sample size: 62 (67.7% male)	Leukemia, Lymphoma, Solid Tumors, Neuroblastoma, CNS Tumors, Sarcoma & Others	Induction/consolidation chemotherapy palliative care	4.5-18 months	Children with low BMI have a poorer prognosis
Jesus Ángel Dominguez-Rojas 2022 (16)	Age: 0-14 years Sample size: 226 (116 men, 110 women)	Blood Cancer and Solid Tumors	radiotherapy bone marrow transplant	7 months	COVID-19 infection increases mortality in cancer patients
Mahmoud Hammad 2021 (17)	Age: median 9 years (range 1-18) Sample size: 76	Acute lymphoblastic leukemia, lymphoma, solid tumors, and others	first-line chemotherapy relapse treatment	2 months	Good clinical outcomes in non-critical patients
Niveditha Balakumar 2022 (18)	Age: 8 days - 18 years Sample size: 45	Uncategorized	palliative care	10 months	The main cause of death is the underlying disease or tumor.
Mariana Cristina M. Corso 2021 (19)	Age: median 6.0 years (range 4-13) Sample size: 179 (M 103, F 76)	leukaemia lymphomas Solid tumors and others	radiotherapy Surgery/radiotherapy	11 months	The mortality rate of children with cancer is significantly higher than that of the general pediatric population

### Quality assessment

A NOS scale was drawn to evaluate the quality of the literature, with NOS scores ranging from 7 to 9 (out of 9). Of the six studies included, all stated that the cases were during the SARS-CoV-2 pandemic, all clinical characteristics and outcomes were objectively collected from medical records, the follow-up period was long enough to meet the requirements to be analyzed, and the study subject selection scores ranged from 3 to 4. All clinical characteristics and outcomes of the studies were objectively collected from medical records, followed up long enough to meet the requirements for the analysis to be performed, and comparable between groups; because some patients in some of the studies were still in the hospital and did not have a final determination of survival or death; and the adequacy of follow-up was not scored independently of the retrospective or prospective design, resulting in an Outcome Measurement Score of 2 to 3. (**Table 2**).

**Table 2:** NOS scale to evaluate literature quality form.

Authors and dates of literature	Selection of study population	Comparability between groups	Outcome measures	Quality assessment score
Collaborators 2022	****	**	***	9
Ana Luiza Magalhães de Andrade-Lima 2023	***	*	***	8
Jesus Ángel Dominguez-Rojas 2022	****	**	**	9
Mahmoud Hammad 2021	***	*	***	7
Niveditha Balakumar 2022	****	*	**	7
Mariana Cristina M. Corso 2021	***	*	***	8

*: one point; **: two points; ***: three points; ****: four points.

### Effects of age on mortality

**Figure 2:**
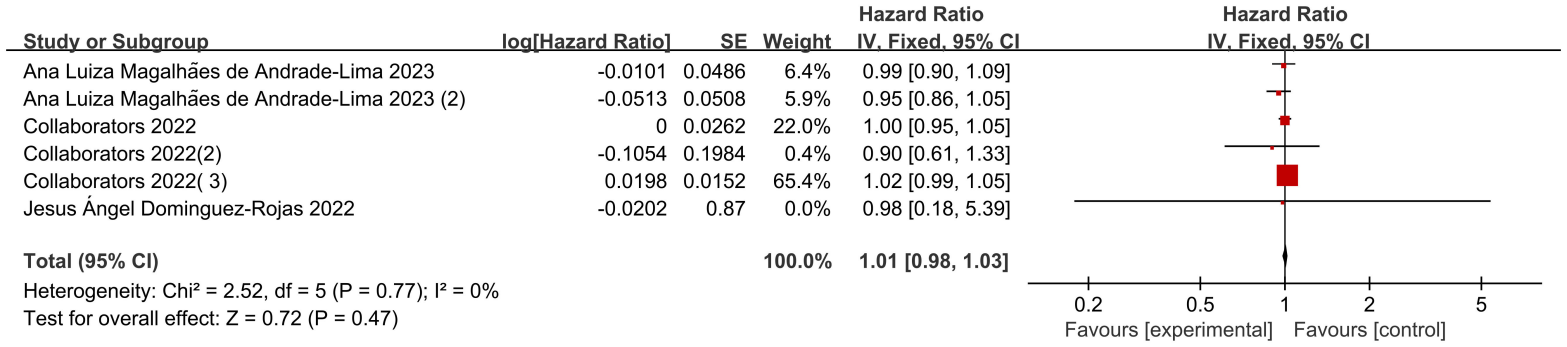
Forest map of age effects on mortality.

Six data sets from three included studies illustrated age-specific mortality-associated hazard ratio (HR) data in pediatric oncology patients after COVID-19 infection. The heterogeneity test showed that there was no heterogeneity in the results (I2 = 0%, P=0.77), so a fixed-effects model was used for Meta-analysis. Meta-analysis showed that mortality in pediatric oncology patients after COVID-19 infection was not associated with age, and that the results were not statistically different [HR=1.01, 95% CI (0.98, 1.03),P=0.47] (**Figure 2**).

### Effects of body weight on mortality

**Figure 3:**
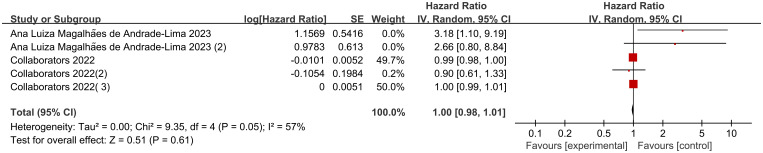
Forest plot of the effect of body weight on mortality.

There were 5 sets of risk ratio (HR) data illustrating the association of different body weights with mortality in pediatric oncology patients after COVID-19 infection in the 2 included studies. Heterogeneity test showed high heterogeneity of results (I2 = 57%, P=0.05), so Meta-analysis was performed using a random effects model.Meta-analysis showed that mortality in pediatric oncology patients after COVID-19 infection was not associated with body weight, and the results were not statistically different [HR=1.00, 95% CI (0.98, 1.01),P= 0.61] (**Figure 3**).

### Effect of clinical complications on mortality

**Figure 4:**
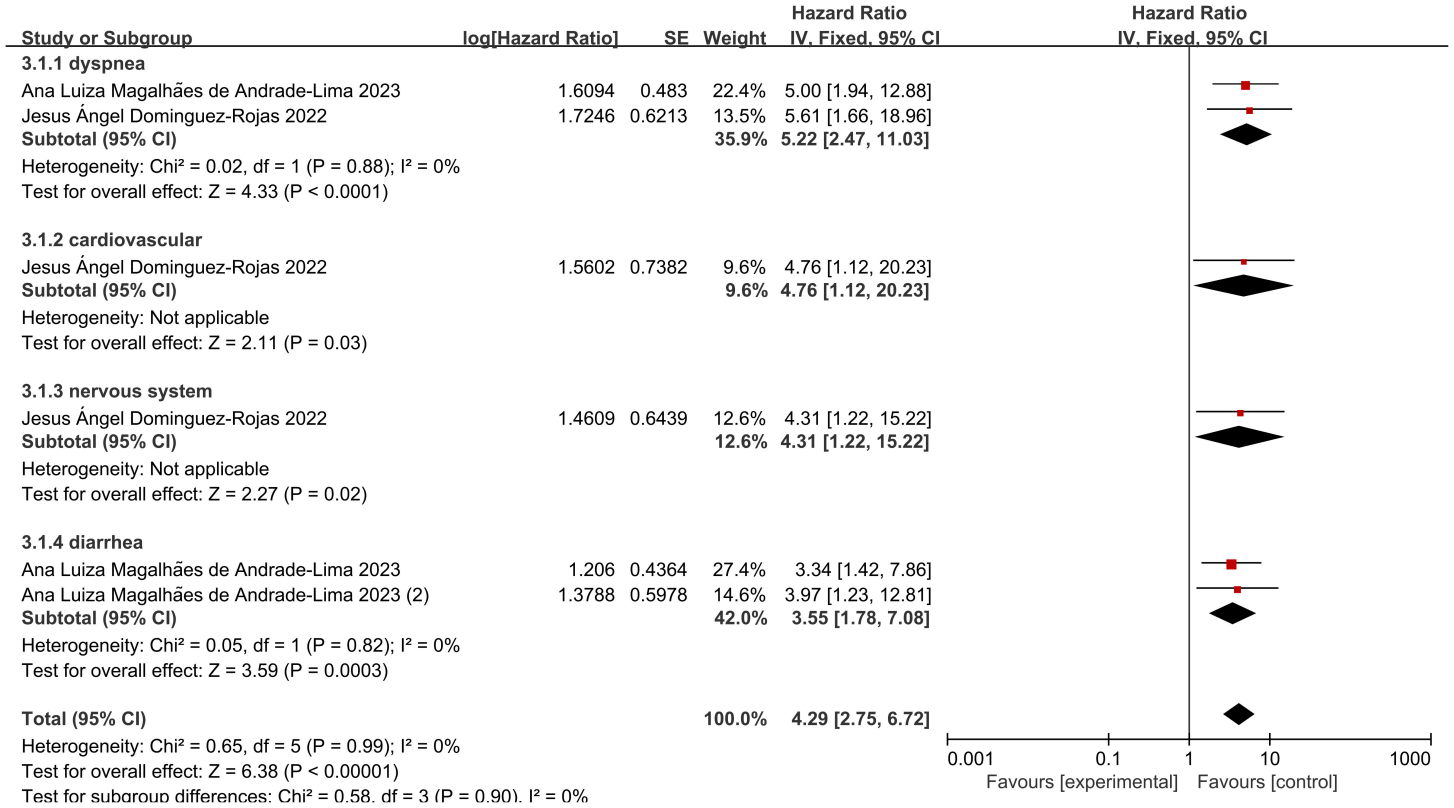
Forest plot of the effect of patient signs on mortality.

Among the two included studies, six groups assessed the hazard ratio (HR) for specific clinical complications (including respiratory distress, cardiovascular complications, neurological complications, and diarrhea) and mortality in pediatric cancer patients following COVID-19 infection. The heterogeneity test showed low heterogeneity of results (I2 = 0%, P = 0.99), and thus a fixed-effects model was used for Meta-analysis. A meta-analysis revealed that the risk of death among pediatric cancer patients following COVID-19 infection was significantly associated with the occurrence of these complications, with statistically significant differences [HR = 4.29, 95% CI (2.75, 6.72), P < 0.00001]. The statistically significant differences indicate that the occurrence of these complications significantly increases mortality. Following screening for different specific clinical complications in pediatric cancer patients, the following complications were identified: respiratory distress, cardiovascular issues, neurological issues, and diarrhea, the analysis of the results revealed that: the heterogeneity of studies presenting with dyspnea was low (I2 = 0%, P = 0.88), and the mortality of pediatric oncology patients after COVID-19 infection was associated with dyspnea, which was a statistically significant difference in the results [HR = 5.22, with a 95% CI (2.47, 11.03),P<0.00001], indicating that the presence of dyspnea in pediatric oncology patients after COVID-19 infection significantly increased mortality; only 1 data showed that the mortality of pediatric oncology patients after COVID-19 infection was associated with cardiovascular conditions, with a statistically significant difference in the results [HR=4.76, 95% CI (1.12, 20.23),P=0.03], suggesting that the presence of cardiovascular conditions significantly increased mortality in pediatric oncology patients after COVID-19 infection; only 1 data showed that mortality in pediatric oncology patients after COVID-19 infection was associated with neurological conditions, with a statistically significant difference in the results [HR=4.31, 95% CI (1.22, 15.22),P=0.02], suggesting that the presence of neurological conditions in pediatric oncology patients after COVID-19 infection significantly increased mortality; the heterogeneity of studies presenting diarrhea was low (I2 = 0%, P=0.82), and the mortality of pediatric oncology patients after COVID-19 infection was associated with diarrhea, with statistically significant results [HR=3.55, 95% CI (1.78, 7.08),P=0.0003], suggesting that the presence of diarrhea in pediatric oncology patients after COVID-19 infection significantly increases mortality. After screening for different clinical complications in pediatric cancer patients, there were no statistically significant differences in respiratory distress, cardiovascular status, neurological status, and diarrhea (P=0.90), indicating that there are differences between different clinical complications (**Figure 4**).

### Impact of acute and critical illnesses on mortality

**Figure 5:**
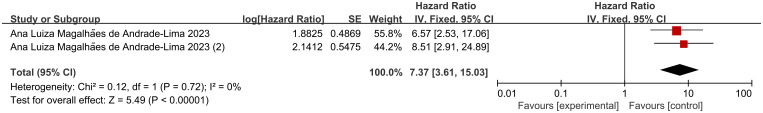
Forest plot of the impact of acute and critical illnesses on mortality rates.

**Figure 6:**
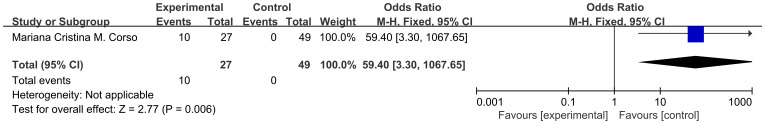
Forest plot of the impact of acute and critical illnesses on mortality rates.

Patients with conditions such as ICU admission, mechanical ventilation, shock, or MODS are defined as “critically ill,” while those with rapidly progressing conditions requiring hospitalization but not meeting the criteria for critical illness are defined as “acutely ill” (41). Two sets of data from 1 included study illustrated risk ratio (HR) data related to mortality in pediatric oncology patients presenting with acute and critical illnesses after COVID-19 infection, and dichotomous variable data comparing mortality in patients with acute and critical illnesses versus those with less severe disease course were present in 1 study. According to **Figure 5**, the heterogeneity test showed that there was no heterogeneity in the results (I2 = 0%, P=0.72), and therefore a fixed-effects model was used for Meta-analysis. Meta-analysis showed that mortality in pediatric oncology patients after COVID-19 infection was associated with acutely ill conditions, with statistically significant results [HR=7.37, 95% CI (3.61. 15.03),P<0.00001]; according to **Figure 6**, the mortality of pediatric tumor patients after COVID-19 infection was associated with acute and critical conditions, with statistically different results [OR=59.40, 95% CI (3.30, 1067.65), P<0.00001], indicating that pediatric cancer patients who developed critical illness after COVID-19 infection had a higher mortality rate than those who did not develop critical illness. Both groups demonstrated a significant increase in mortality in acutely ill pediatric oncology patients after COVID-19 infection.

### Effects of cancer type on mortality

**Figure 7:**
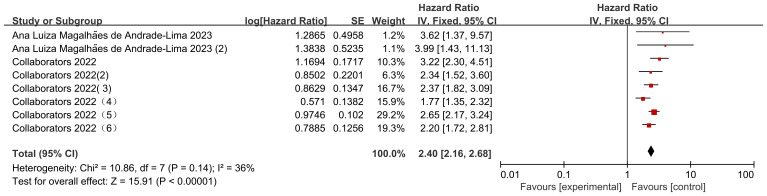
Forest plot of the effect of cancer type on mortality.

There were 8 data sets from the 2 included studies illustrating risk ratio (HR) data related to mortality from solid versus non-solid tumors in pediatric oncology patients after COVID-19 infection. The heterogeneity test showed that there was low heterogeneity of results (I2 = 36%, P = 0.14), so a fixed-effects model was used for Meta-analysis. Meta-analysis showed that mortality rates of solid tumors were higher than those of non-solid tumors in pediatric oncology patients after COVID-19 infection, with a statistically significant difference in the results [HR = 2.40, 95% CI (2.16, 2.68), P < 0.00001], indicating that solid tumors significantly increased mortality in pediatric oncology patients with COVID-19 infection (**Figure 7**).

**Figure 8:**
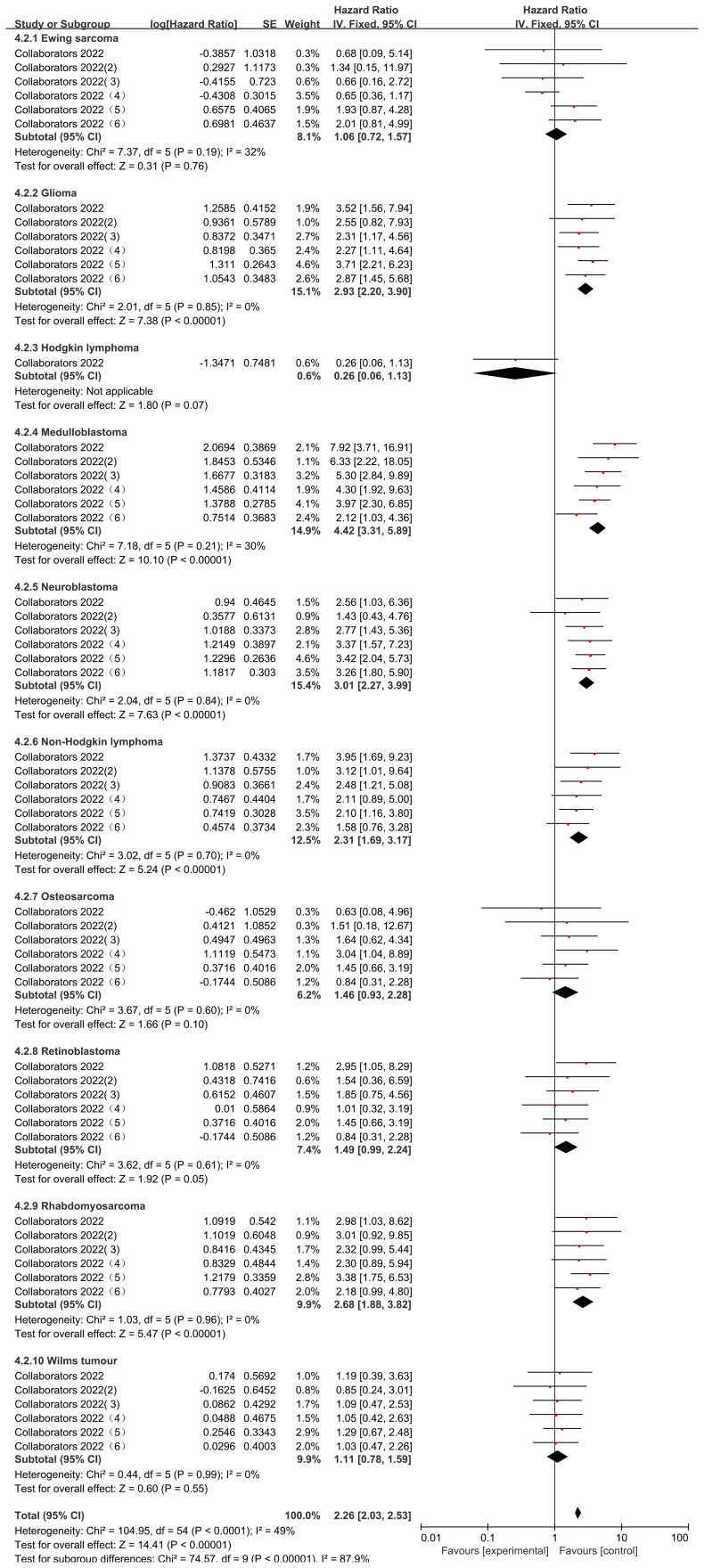
Impact of specific cancer types on mortality.

The Collaborators 2022 study reported risk ratio (HR) data related to the occurrence of mortality for different types of childhood oncology cancers compared to hematologic cancers during the observation period. Only common cancers were analyzed in this study including Ewing sarcoma, Glioma, Hodgkin lymphoma, Medulloblastoma, Neuroblastoma, Non-Hodgkin lymphoma, Osteosarcoma, Retinoblastoma, Rhabdomyosarcoma, Wilms tumour. heterogeneity was low in all studies (Ewing sarcoma (I2 = 32%, P=0.19), Glioma (I2 = 0%, P=0.85), Medulloblastoma (I2 = 30%, P=0.21), Neuroblastoma (I2 = 0%, P=0.84), Non-Hodgkin lymphoma (I2 = 0%, P=0.70), Osteosarcoma (I2 = 0%, P=0.60), Retinoblastoma (I2 = 0%, P=0.61), Rhabdomyosarcoma (I2 = 0%, P=0.96), and Wilms tumour (I2 = 0%, P=0.99), and were therefore analyzed using a fixed effects model. Those that did not differ from the blood group of cancers after using the comparison were Ewing sarcoma (HR=1.06, 95% CI (0.72, 1.57), P=0.76), Hodgkin lymphoma (HR=0.26, 95% CI (0.06, 1.13), P=0.91), Osteosarcoma (HR= 1.46, 95% CI (0.93, 2.28), P=0.86), Retinoblastoma (HR=1.49, 95% CI (0.99, 2.24), P=0.05), Wilms tumour (HR=1.11, 95% CI (0.78, 1.59), P=0.55), and in the case of Glioma (HR=2.93, 95% CI (2.20, 3.90), P<0.00001), Medulloblastoma (HR=4.42, 95% CI (3.31, 5.89), P<0.00001), Neuroblastoma (HR=3.01, 95%CI (2.27, 3.99), P < 0.00001), Non-Hodgkin lymphoma (HR = 2.31, 95% CI (1.69, 3.17), P < 0.00001; Figure), Rhabdomyosarcoma (HR = 2.68, 95% CI (1.88, 3.82), P < 0.00001) mortality rates were significantly higher than those for hematologic cancers. There was a statistically significant difference in the post-screening comparison of different cancer deaths in pediatric oncology patients (P < 0.00001), indicating significant variability between different cancer mortality rates (**Figure 8**).

### Effect of cancer treatment on mortality

**Figure 9:**
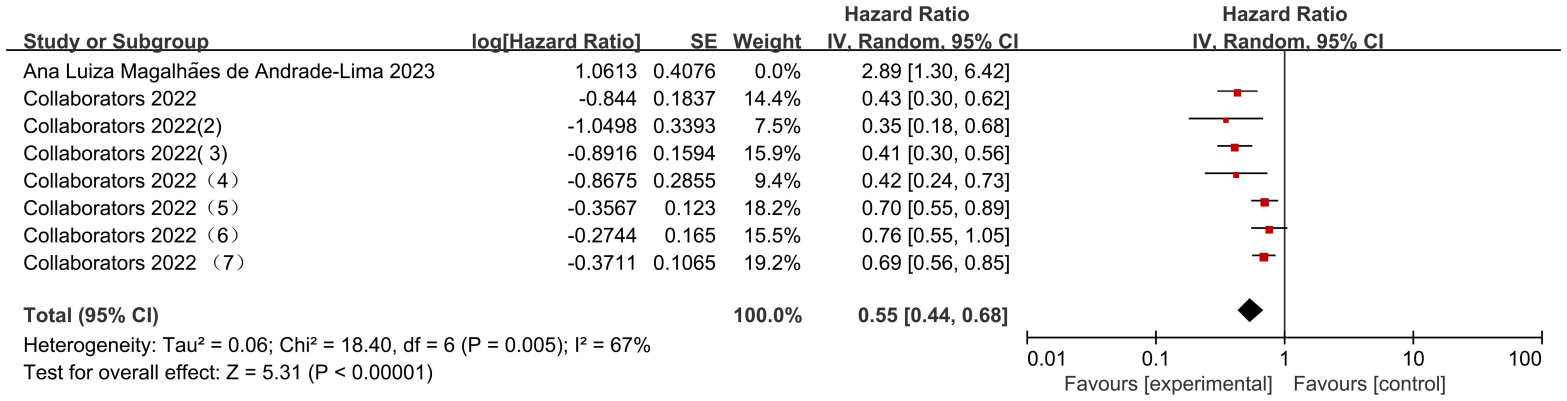
Forest plot of the impact of cancer treatment on mortality.

Seven data sets from the 2 included studies illustrate the risk ratio (HR) data associated with mortality in pediatric oncology patients with cancer consolidation therapy after COVID-19 infection. The heterogeneity test showed high heterogeneity of results (I2 = 67%, P = 0.005), and therefore a random-effects model was used for Meta-analysis. Meta-analysis showed statistically significant differences in the results of mortality associated with cancer consolidation therapy in pediatric oncology patients after COVID-19 infection [HR = 0.55, 95% CI (0.44, 0.68), P < 0.00001], indicating that pediatric cancer patients with COVID-19 infection who received active cancer consolidation therapy had significantly lower mortality rates than those who did not receive treatment or received less intensive treatment (**Figure 9**).

**Figure 10:**
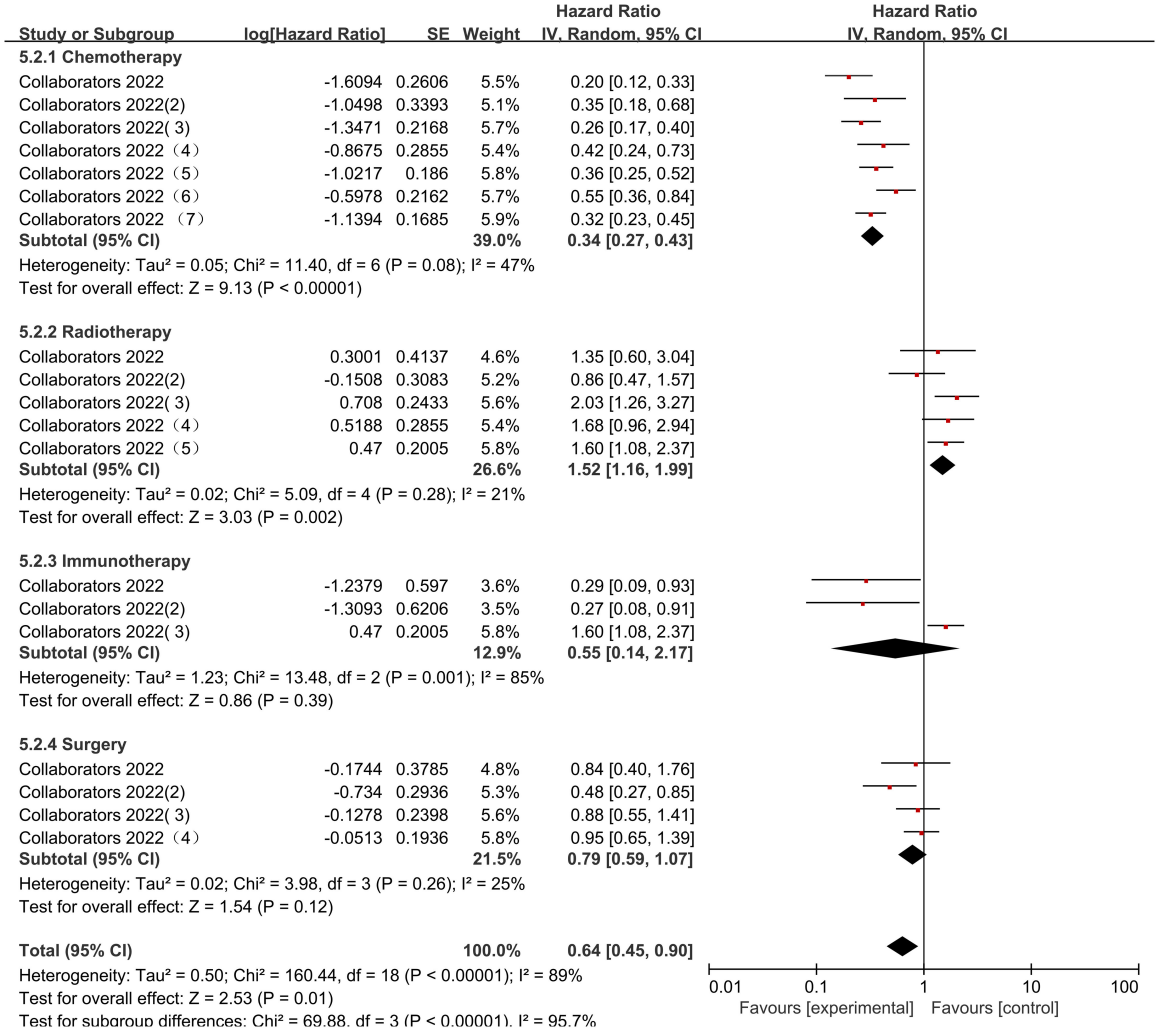
Impact of different types of cancer treatment on mortality.

The Collaborators 2022 study reported risk ratio (HR) data related to the occurrence of mortality for different cancer consolidation treatments performed during the observation period. Only common cancer consolidation treatments including Chemotherapy, Radiotherapy, Immunotherapy, and Surgery were analyzed in this study. There was high heterogeneity in the final outcome of the studies (I2 = 89%, P<0.00001), so all were analyzed using a random effects model. The heterogeneity in the chemotherapy studies was low (I² = 47%, P = 0.08). Patients who received chemotherapy after COVID-19 infection had a significantly lower mortality rate than those who did not receive chemotherapy, with statistically significant differences [HR = 0.34, 95% CI (0.27, 0.43), P < 0.00001]; Studies on radiotherapy showed low heterogeneity (I² = 21%, P = 0.28). Patients who received radiotherapy after COVID-19 infection had a significantly higher mortality rate than those who did not receive radiotherapy, with statistically significant differences [HR = 1.52, 95% CI (1.16, 1.99), P = 0.002]; there was high heterogeneity of studies that performed Immunotherapy (I2 = 85%, P=0.001), COVID-19 infection was not associated with Immunotherapy and the results were not statistically different [HR=0.55, 95% CI (0.14, 2.17),P=0.39]; there was low heterogeneity in studies that performed Surgery (I2 = 25%, P=0.26), and deaths of pediatric tumor patients with COVID-19 infection were not associated with Surgery and the results were not statistically different [HR=0.79, 95% CI (0.59, 1.07),P=0.12]. There was a statistical difference between the post-surgery comparisons of consolidation therapy deaths for different cancers in pediatric oncology patients (P < 0.00001), indicating significant variability between consolidation therapy mortality rates for different cancers (**Figure 10**).

### Subgroup analysis of cancer types

**Figure 11:**
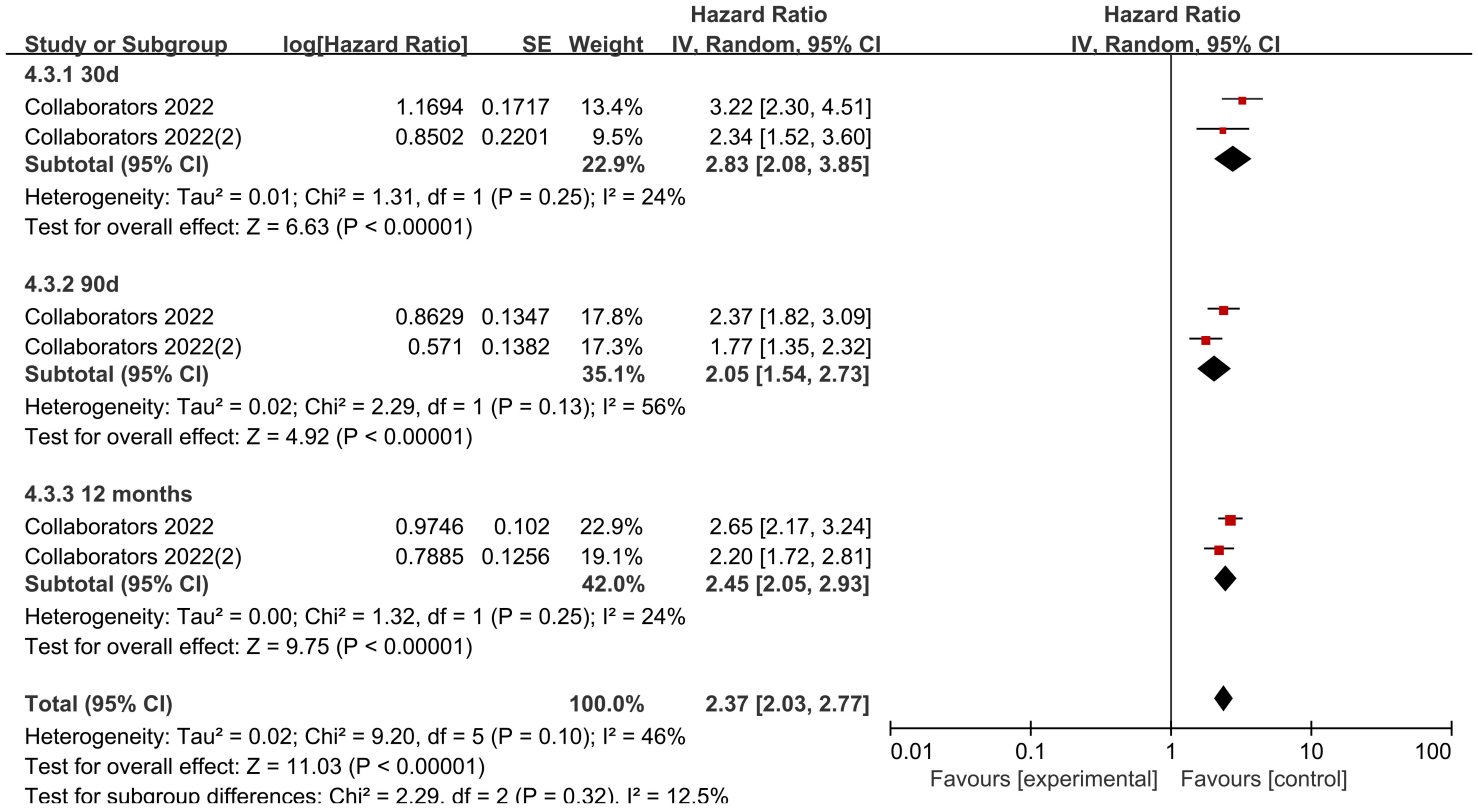
Subgroup analysis of the effect of different cancer types on mortality.

Looking at the comparison of solid tumor and non-solid tumor mortality rates by data according to different follow-up times, the heterogeneity of the studies with a follow-up time of 30 d was low (I2 = 24%, P=0.25), and the mortality rate of solid tumors was higher than that of non-solid tumors in pediatric oncology patients after COVID-19 infection, with statistically significant differences in the results [HR=2.83, 95% CI (2.08, 3.85),P< 0.00001]; the heterogeneity of studies with a follow-up time of 90 d was high (I2 = 56%, P=0.13), and the mortality rate of solid tumors was higher than that of non-solid tumors in pediatric oncology patients after COVID-19 infection, with statistically different results [HR=2.05, 95% CI (1.54, 2.73),P<0.00001]; the heterogeneity of studies with a follow-up time of 12 months was was high (I2 = 24%, P=0.25), and the mortality rate of solid tumors was higher than that of non-solid tumors in pediatric oncology patients after COVID-19 infection, and the results were statistically different [HR=2.45, 95% CI (2.05, 2.93),P<0.00001]. Comparison of differences between groups showed that there was no difference in comparison of different follow-up times in pediatric tumor patients (P=0.32), which could not indicate that follow-up time was a source of heterogeneity affecting the results (**Figure 11**).

### Subgroup analysis of cancer consolidation therapy

**Figure 12:**
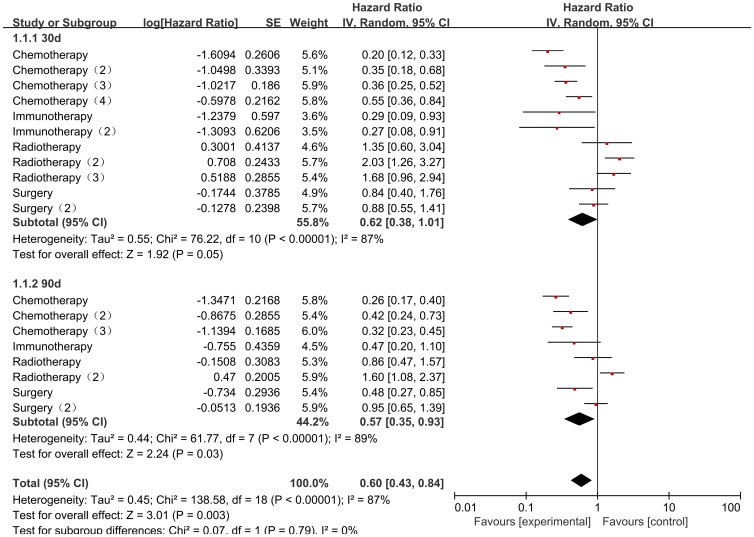
Subgroup analysis of consolidation therapy for different types of cancer.

When comparing cancer consolidation therapy according to different follow-up durations, there was a high degree of heterogeneity in both follow-up durations of 30 d (I2 = 24%, P=0.25) and 60 d (I2 = 24%, P=0.25). Results of subgroup analysis: the mortality outcome of cancer consolidation therapy in pediatric oncology patients with a follow-up time of 30d was not statistically different [HR=0.62, 95% CI (0.38, 1.01),P=0.05], whereas the mortality rate of cancer consolidation therapy was lower with a follow-up time of 60d, with a statistically significant difference in the outcome [HR=2.83, 95% CI (2.08. 3.85),P<0.00001]; however, in terms of more between-group differences, there was no difference in the comparison of different follow-up times in pediatric oncology patients (P=0.79), and it was not possible to show that the follow-up time was a source of heterogeneity affecting the results (**Figure 12**).

### Sensitivity analysis

In this study, sensitivity analyses were conducted by systematically assessing the sources of heterogeneity, and the fixed-effects model was compared with the random-effects model for outcome indicators with different levels of heterogeneity to test the robustness of the combined results. The results showed that when the effect model was switched, the estimates of the combined effect sizes of the risk factors were highly consistent (the results of the fixed-effects model were within the 95% confidence interval of the random-effects model), and the direction and magnitude did not change substantially, indicating that the results of the Meta-analysis were insensitive to the choice of model, and the overall results showed a low sensitivity and a high level of robustness. This result further supports the reliability of the main findings and suggests that potential confounders have limited influence on effect sizes (**Table 3**).

**Table 3:** Sensitivity analysis table.

Outcome indicator	Effect model	Effect size	95 per cent confidence interval	Effect model	Effect size	95 per cent confidence interval
Age	FE	HR=1.01	(0.98,1.03)	RE	HR=1.01	(0.98,1.03)
Weight	FE	HR=1.00	(0.99,1.00)	RE	HR=1.00	(0.98,1.01)
Dyspnea	FE	HR=5.22	(2.47,11.03)	RE	HR=5.22	(2.47,11.03)
Cardiovascular system	FE	HR=4.79	(1.12,20.23)	RE	HR=4.79	(1.12,20.23)
Nervous System	FE	HR=4.31	(1.22,15.22)	RE	HR=4.31	(1.22,15.22)
Diarrhea	FE	HR=3.55	(1.78,7.08)	RE	HR=3.55	(1.78,7.08)
Solid tumors	FE	HR=2.40	(2.16,2.68)	RE	HR=2.42	(2.09,2.80)
Ewing sarcoma	FE	HR=1.06	(0.72,1.57)	RE	HR=1.12	(0.66,1.89)
Glioma	FE	HR=2.93	(2.20,3.90)	RE	HR=2.93	(2.20,3.90)
Hodgkin lymphoma	FE	HR=0.26	(0.06,1.13)	RE	HR=0.26	(0.06,1.13)
Medulloblastoma	FE	HR=4.42	(3.31,5.89)	RE	HR=4.46	(3.14,6.35)
Neuroblastoma	FE	HR=3.01	(2.27,3.99)	RE	HR=3.01	(2.27,3.99)
Non-Hodgkin lymphoma	FE	HR=2.31	(1.69,3.17)	RE	HR=2.31	(1.69,3.17)
Osteosarcoma	FE	HR=1.46	(0.93,2.28)	RE	HR=1.46	(0.93,2.28)
Retinoblastoma	FE	HR=1.49	(0.99,2.24)	RE	HR=1.49	(0.99,2.24)
Rhabdomyosarcoma	FE	HR=2.68	(1.88,3.82)	RE	HR=2.68	(1.88,3.82)
Wilms tumour	FE	HR=1.11	(0.78,1.59)	RE	HR=1.11	(0.78,1.59)
Cancer consolidation therapy	FE	HR=0.59	(0.53,0.69)	RE	HR=0.55	(0.44,0.68)
Chemotherapy	FE	HR=0.34	(0.29,0.40)	RE	HR=0.34	(0.27,0.43)
Radiotherapy	FE	HR=1.54	(1.22,1.94)	RE	HR=1.52	(1.16,1.99)
Immunotherapy	FE	HR=1.17	(0.82,1.67)	RE	HR=0.55	(0.14,2.17)
Surgery	FE	HR=0.81	(0.63,1.04)	RE	HR=0.79	(0.59,1.07)
acute and critical illness	FE	HR=7.37	(3.61,15.03)	RE	HR=7.37	(3.61,15.03)

FE: fixed effects model; RE: random effects model.

## Publication bias

The inverted funnel plots of age, body weight, different clinical complications, solid tumor status and cancer consolidation treatment were plotted as independent variables, and it can be seen that most of the studies on age and solid tumor were in the upper part of the inverted funnel, while there were fewer studies in the bottom part of the inverted funnel, and the symmetry was roughly between the left and right sides, which suggested that there was no obvious publication bias. Most of the studies on body weight, different clinical complications, and cancer consolidation therapy were in the middle of the “inverted funnel” and symmetrical, suggesting that publication bias might exist. For the rest of the outcome indicators, funnel plots were not created to observe publication bias because of the small amount of data. For details, see **Supplementary Material 1**.

## Discussions

Early reports (21, 22) at the beginning of the covid pandemic identified a variety of factors contributing to the poorer prognosis of patients with covid, including advanced age, cardiac insufficiency, respiratory disease, and cancer. Unusually, in the present study we pooled deaths in COVID-19 pediatric oncology patients by age and found that age did not show a large difference compared to adult patients, one explanation for this being that incomplete maturation of adaptive immunity incomplete maturation may protect children from excessive inflammation in adults (23). Data suggesting (24) that children and young adults with cancer who are overweight/obese or Hispanic/Latino do not seem to be at higher risk for COVID-19 adverse outcomes compared to other pediatric oncology patients validate the conclusions obtained in our summary.

Cardiovascular complications of COVID-19, such as cardiac injury, heart failure, and arrhythmias, were more prevalent in patients who died than in those who survived. Of these, cardiac injury is associated with the highest risk of death and has been extensively studied. Possible mechanisms by which COVID-19 causes cardiac injury include cardiac stress due to respiratory failure and hypoxemia, direct infection of the myocardium by viruses, and indirect injury due to systemic inflammatory responses (25). The data from the studies we included identified adverse physical complications in pediatric patients after COVID-19 all demonstrated higher mortality, both in terms of cardiovascular, respiratory, gastrointestinal symptoms, and neurologic aspects. In general, COVID-19 patients may experience adverse complications such as fever, cough, muscle aches, fatigue, headache, gastrointestinal symptoms, and difficulty breathing (26). Among these symptoms, dyspnea was significantly associated with increased mortality in pooled analyses, confirming the findings of two studies (27, 28) that dyspnea was associated with increased mortality, even after adjusting for age, sex, and other confounders. Because dyspnea is readily observed in clinical practice, it may be a valuable predictor in identifying individuals who are at high risk for fatal outcomes and may require additional attention.

Previous studies (29) have found a wide variation in mortality after covid infection in cancer patients, ranging from 9% to 33%, and no clear association was found between anticancer therapy, cancer characteristics, and mortality from covid infection. Immunocompromised pediatric patients, especially cancer patients, are a susceptible population for COVID-19 and should be closely followed up by healthcare professionals (30). However, there is no specific treatment for pediatric cancer patients infected with COVID-19. In this regard, a global report presented by the Cancer Society consolidated essential information for clinicians regarding the diagnosis and treatment of COVID-19 in cancer patients (31). The report recommends that both children and care teams wear effective personnel protective equipment to prevent the spread of COVID-19. In addition, the report encourages children undergoing surgical treatment for cancer not to delay surgery during a COVID-19 epidemic to improve disease prognosis. In addition, identifying the source of infection and transmission dynamics (either by patients or medical staff) in each oncology center is essential to prevent new COVID-19 cases (31). In addition, it has been suggested that pediatric cancer patients infected with SARS-CoV-2 should not undergo major adjustments to their primary therapy (30). In this regard, when we summarized the data on cancer consolidation therapy, we analyzed different treatment follow-up times, and active treatment improved the prognosis of patients after a long follow-up period, whereas there was no difference in the earlier period. This meta-analysis categorized the means of consolidation of cancer treatment, and although, overall, active treatment was effective in improving the prognosis of death, the categorization improved the prognosis of patients only in the case of chemotherapy, but not in the case of immunotherapy and surgery, and increased the risk of death in the case of radiotherapy. Given the risk of COVID-19 infection worsening with immunosuppressive anticancer as well as radiation therapy, and the fact that COVID-19 infection in cancer patients can have a significant impact on effective anticancer therapy, we hypothesized that while incompletely matured adaptive immunity in pediatric patients can reduce symptoms of hyperinflammation, it can also affect their own healthy immune systems, thereby worsening the prognosis of the child.

When we pooled the data from our study, we found that acutely ill COVID-19 pediatric cancer patients had a worse prognosis for death, with a substantially lower mortality rate than patients with milder symptoms. This is consistent with other studies (32–34), where the overall proportion of deaths was lower for severe/critical infections and those requiring intensive care. Although the rate of severe/critical infections was low for the entire cohort, CAR-T recipients of COVID-19 tended to develop severe/critical infections, which is consistent with the high risk of COVID-19 results for adverse outcomes in the context of CAR-T-induced B-cell immunodeficiency (35).

Finally, for the different types of cancer, we broadly differentiated into two main categories, hematologic and solid tumors, and compared the various types of tumor types. Hematologic tumors are the most common tumors in pediatrics and are more severe and have a higher mortality rate due to complications, including infections, compared to solid tumors (37–39). Perhaps for this reason, more hematological malignancy patients died during hospitalization for COVID-19, while solid tumor patients had a higher risk of death throughout the follow-up period.

In summary, compared to other common viral or bacterial infections faced by pediatric cancer patients (such as influenza, RSV, or sepsis), COVID-19 infection exhibits unique mortality risk characteristics. Its pathophysiological mechanisms, particularly the significant hypercoagulable state/thrombotic tendency and complex immune dysregulation (such as early lymphopenia and potential excessive inflammation), interact with cancer- and treatment-related immunosuppression, potentially leading to unique fatal complication patterns (such as pulmonary embolism and specific organ damage). While severe respiratory failure and septic shock are common causes of death in various severe infections, this study found that patients with solid tumors and specific complications (such as dyspnea or cardiovascular events) are at particularly high risk. Additionally, the unprecedented strain on medical resources and treatment delays caused by the COVID-19 pandemic have led to indirect cancer-related deaths, representing a significant and important “indirect” mortality impact distinct from other infections. Although direct comparisons of mortality rates between different pathogens are challenging (e.g., study design, patient population, differences in supportive care), COVID-19, due to its extremely high transmissibility and pandemic scale, poses a significant overall burden on the pediatric cancer population. The risk factors identified in this study provide a basis for clinical risk stratification under this specific threat.

Strengths of this systematic review and Meta-analysis-. The first more systematic de- analyzed risk factors for eventual death after COVID-19 infection in pediatric oncology patients, the analysis included strict inclusion and exclusion criteria, and the methodological quality of the study was assessed. The data were statistically studied using the NOS quality rating scale, subgroup analysis, sensitivity analysis, and publication bias, and addressed the fact that previous meta-analysis (36) did not differentiate between solid tumors and other malignant tumors, and gained insights into how to rationally design the treatment of COVID-19 late cancer therapy and attention to patients with acute and severe illnesses.

Our meta-analysis shows that children with cancer who were infected with COVID-19 and received active cancer consolidation therapy (particularly chemotherapy) had significantly lower mortality rates, while those who received radiotherapy were associated with higher mortality rates. However, it is important to emphasize that these findings represent observed associations and do not establish causality. There are several important confounding factors that may influence these results. For example, patients who received active chemotherapy are typically more closely monitored, are more likely to be hospitalized, or receive intensive care in medical facilities. This earlier disease identification, more timely COVID-19 diagnosis, and more intensive supportive care, rather than chemotherapy itself, may be the primary reasons for the lower mortality rate in this group. Additionally, patients who were able to continue receiving active treatment may represent a group with better baseline health status, greater tolerance for cancer treatment, or relatively milder COVID-19 infection severity (selection bias). Conversely, the group of patients requiring radiotherapy may have specific tumor types or more advanced disease stages, which are themselves associated with poorer outcomes. The need for radiotherapy may serve as a marker of disease severity rather than a direct cause of mortality. Similarly, the observed lack of significant association between immunotherapy and surgery and mortality may be influenced by small sample sizes, selection bias, and uncontrolled confounding factors. Therefore, these results should be interpreted as descriptive associations, suggesting the need for further research (such as prospective studies with detailed adjustment for confounding factors or propensity score matching analysis) to clarify the true impact of cancer treatment in the context of COVID-19 infection. Clinical decisions should comprehensively consider the individual patient’s cancer status, COVID-19 severity, and potential treatment risks and benefits, rather than relying solely on these observational association results.

There are limitations- of this study. In our Meta-analysis. Firstly, the data of the study was small and all European and American populations, and there was no East Asian population for refinement, which may affect the new conclusions obtained. Secondly, due to the limited information provided in the included studies, there are still many potential predictors of mortality that could not be extracted and summarized. Third, the current meta-analysis suffers from large heterogeneity in outcome indicators, and these heterogeneities can, although they can be explained by differences in patient populations and disease severity, which we were unable to perform. Fourth, the quality of included studies varied widely. More high-quality studies are needed for further analysis.

## Data Availability

The datasets presented in this study can be found in online repositories. The names of the repository/repositories and accession number(s) can be found below: OSF:https://osf.io/tfz9r/?view_only=eac4c54f75af4834848e691f03aa9788.
