# Supplementary figures and images for: Meta-analysis of mortality factors after COVID-19 infection in pediatric oncology patients
Source: Front Oncol. 2025 Aug 6;15:1594617. doi: 10.3389/fonc.2025.1594617 (PMC12364703; doi:10.3389/fonc.2025.1594617)

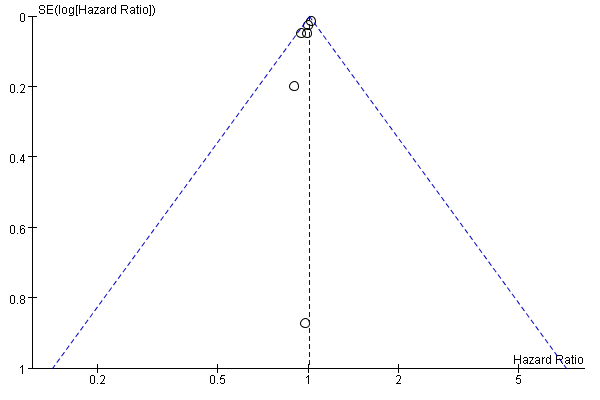


Age
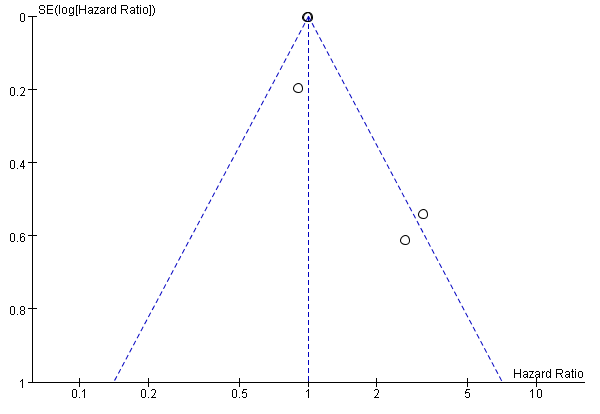


Weight
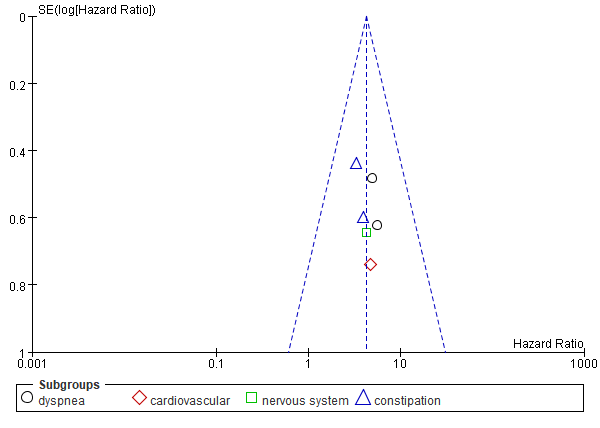


signs
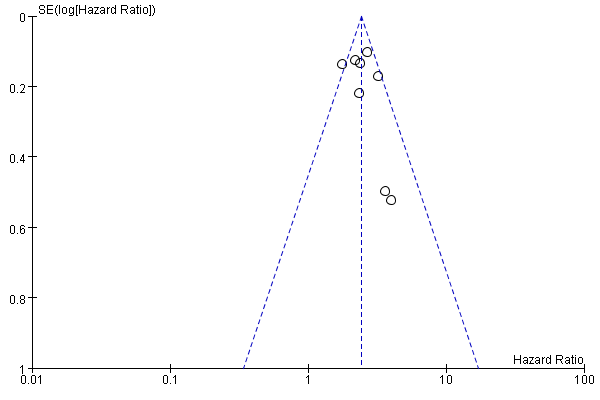


solid tumor
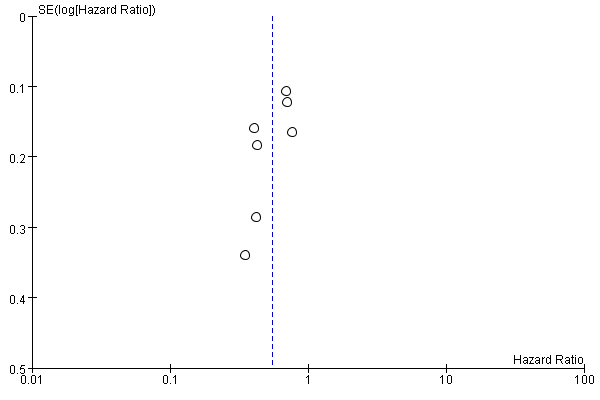


Cancer consolidation therapy

Supplement: Supplementary file 1 [file DataSheet1.docx]
